# Alcohol use disorders after bariatric surgery: a study using linked health claims and survey data

**DOI:** 10.1038/s41366-024-01606-3

**Published:** 2024-09-06

**Authors:** Oliver Riedel, Malte Braitmaier, Mark Dankhoff, Ulrike Haug, Melanie Klein, Wiebke Zachariassen, Jana Hoyer

**Affiliations:** 1 Leibniz Institute for Prevention Research and Epidemiology—BIPS, Bremen, Germany; 2 Hamburg, Germany; 3 University of Bremen, Faculty of Human and Health Sciences, Bremen, Germany; 4 DAK Gesundheit, Hamburg, Germany; 5 Adipositas-Zentrum, Städtisches Klinikum Dresden, Dresden, Germany

**Keywords:** Lifestyle modification, Obesity

## Abstract

**Background:**

Previous studies have repeatedly reported alcohol use disorders (AUDs) in patients after bariatric surgery (BS). This research field can benefit from studies combining health claims data with survey data.

**Methods:**

Based on a combined retrospective cohort and cross-sectional study, 2151 patients with BS identified in a large health claims database received a questionnaire, by which we assessed the presence of AUDs based on a validated instrument (AUDIT) as well as by ICD-10 codes from the health claims data. We described patients with vs. without AUDs regarding sex, time since surgery, satisfaction with weight loss and health care resource utilization (HCRU).

**Results:**

The majority of patients were female (80.7%) with a median time since surgery of 6 years (Interquartile range: 4–9 years). For the majority of patients, the bariatric intervention was either a RYGB-Bypass (50%) or sleeve gastrectomy (43%). Overall, 3% had at least one AUD diagnosis code in the claims data (men: 5.5%, women: 2.5%). Among men, 43.6% of diagnoses were coded after but not before the surgery (women: 52%). According to AUDIT (completed by 1496 patients), 9.4% of all patients showed at least hazardous/harmful alcohol consumption. Higher scores were associated with sex of the person, longer time since surgery, dissatisfaction with the weight loss and higher HCRU, with contradicting results regarding psychotherapeutic care.

**Conclusions:**

The proportion with AUDs in the study population gives rise to concern as alcohol consumption should be restricted after BS. The results suggest the necessity for close monitoring and post-surgical care.

## Background

There is evidence that severe obesity and addictive disorders are potentially connected or even causally related [1, 2], partly with inconsistent findings between men and women [3]. The relationship between obesity and alcohol disorders is especially relevant for patients living with severe overweight. Bariatric surgery (BS) —indicated in patients with a body mass index (BMI) of more than 40 kg/m² after unsuccessful conventional therapy—permanently modifies the anatomy of the gastrointestinal tract by bypassing the digestive tract. This also affects the digestion of alcohol: it is absorbed more rapidly, higher maximum concentrations are reached, and the elimination time is prolonged, increasing the risk of addiction and alcohol-related harm [4–6]. Therefore, alcohol use disorders (AUDs) are a contraindication for BS [7]. Data suggest that patients undergoing BS are subsequently at increased risk of developing substance use disorders, particularly AUDs [8] but also abuse of other drugs [9, 10]. Despite variations in the frequencies of AUDs in patients after BS reported in the literature, the review by Linlin and Wu [11] summarized a prevalence in the range of 8–18% shortly after surgery and up to 35% as a lifetime prevalence. Up to 43% of all patients with post-surgical AUDs did not show any signs of pre-surgical AUDs [12].

In recent years, an increasing number of findings on AUDs in patients with BS have been published, although the results were sometimes inconclusive due to heterogeneous methodological quality [13]. Flaws of some of the previous studies comprise their limitations regarding the available follow-up times or their sample sizes, which did not allow sufficiently stratified analyses of subgroups of patients (e.g. by sex or by type of bariatric intervention). The repeated calls for studies [14, 15] with a longer observation period and larger sample sizes were addressed by a recently published, high-quality study using on health claims data [16]. Based on a follow-up time of more than 8 years and more than 400 000 patients with bariatric interventions, the authors reported an increased post-surgical risk for AUDs according to coded ICD-diagnoses. This research field can further benefit from studies that combine these advantages of health claims data with the advantages of survey data, which can provide a more detailed picture on AUDs by using standardized questionnaires.

Therefore, the present study aims at describing the presence of alcohol-related disorders in 2151 persons with BS before and up to 12 years after surgery, based upon a linked data set comprising health claims data and survey data.

## Methods

### Study design

The design of the underlying “ABARO” study has been presented previously in more detail [17]. Briefly, we linked longitudinal health claims data (cohort analyses) with cross-sectional survey data from patients after BS using a multistep approach. First, we used data from one statutory health insurance (SHI) provider included in the German Pharmacoepidemiological Research Database (12 million persons who have been insured at this SHI since 2004 or later) [18] to identify patients with BS anytime between 2004 and 2018 and who were still alive at the end of 2018 (*n* = 6913). After excluding *n* = 1691 patients who could not be contacted for various reasons (e.g. end of insurance with this SHI or objection to receiving mail), a questionnaire was sent to a total of *n* = 5222 patients in 2021 (see the next section for more details). Hereof, *n* = 2521 patients responded. As for the present analyses, only patients with at least three years of baseline prior to surgery were considered, *n* = 267 patients without sufficient baseline were excluded. For *n* = 103 patients, surveys could not be linked to the claims data due to lack of consent. Therefore, the analysis data set comprised *n* = 2151 patients with BS between 2007 and 2018, for whom both cross-sectional survey and longitudinal health claims data were available (see Supplementary Fig. S1 for further details). For the cohort analyses in this paper, the time prior to the BS was considered as “pre-surgery”, the time after surgery as “post-surgery”.

### Assessment of AUDs

The study variables were derived from the health claims data and survey data.

Based on health claims data, AUDs were assessed by the existence of corresponding diagnostic ICD-10 codes (F10). To avoid overestimation, AUDs were only considered if they were documented by at least (a) one inpatient main or secondary discharge diagnosis, or (b) two (or more) identical outpatient diagnoses in subsequent quarters or (c) two (or more) outpatient diagnoses in the same quarter coded by different physicians. In sensitivity analyses, we only considered patients with at least one inpatient discharge diagnosis of AUDs (additional outpatient diagnoses were allowed) as inpatient diagnoses typically have the highest validity. Each patient was further categorized as to whether they had AUDs only pre-surgery, only post-surgery, pre- and post-surgery (“both”) or never.

In the survey, AUDs were measured with the Alcohol Use Disorders Identification Test (AUDIT), which consists of 10 items addressing aspects of alcohol consumption (e.g. frequency of consumption, loss of control). The value of each item ranges from 0 ( = unproblematic) to 4 ( = severe), thus constituting a possible total score between 0 and 40. Based on this total score, patients were categorized as having “low-risk consumption” (scores ≤7), “hazardous/harmful consumption” (scores 8–14) or a “moderate-severe alcohol use disorder” (scores ≥15) [19, 20]. As these cut-off scores have not been validated in patients after BS before, we have additionally conducted three sensitivity analyses as presented in Suppl. Table S1 to avoid underestimations of AUDs in this population. In analysis A, we doubled the weighting of the consumption items 1–3; in analysis B, we increased the score of these items by one and in analysis C, we chose lower cut-offs for the total score categories as mentioned above (≤4, 5–11 and ≥12).

### Further study variables

Comorbidities were assessed using the health claims data, implementing algorithms of high specificity by taking into account either inpatient codes or information on medication [21]. The anthropometrics were derived from the survey data. The BMI was calculated by dividing the body weight (in kilograms) by the square of height in meters, and was categorized as “normal weight” (18.5–24.9), “overweight” (25.0–29.9), “obesity class I” (30.0–34.9), “obesity class II” (35.0–39.9) and “obesity class III” (>40). The total weight loss (TWL, %) was calculated by dividing the number of kilograms lost by the number of kilograms in the patient’s pre-surgical body weight. The utilization of health care services (i.e. number of inpatient treatment days, hospitalizations, outpatient visits and psychosocial/psychotherapeutical interventions) was estimated by health claims data and survey data.

### Statistical analyses

Summary statistics consisted of counts and percentages. Means and medians are presented with 95% confidence intervals (CI) and quartiles (Q1, Q3), where appropriate. Based on previous recommendations and debates [22–24], we did not perform significance tests. First, because this observational study was not based on specific hypotheses and secondly to avoid misinterpretation of *p* values due to the large sample size. Associations with categorical predictor variables were calculated by using odds ratios (OR) estimated from logistic regressions with 95% CI. All statistical analyses were conducted using *R* version 4.3.1 and SAS 9.4 (program codes available on request).

### Ethics

The study was approved by the Hamburg Medical Chamber Ethics Committee (October 11, 2021, No. 2021-10543-BO-ff) and was performed according to the 1964 Declaration of Helsinki. Written informed consent was obtained from each participant.

## Results

### Study population

The *n* = 2151 included patients had a mean age of 54.6 years (95% CI: 54.1—55.0) when they filled the survey, and 80.7% were women. The median time since surgery was 6 years (Q1: 4; Q3: 9). The mean BMI at the conduction of the survey was 34.8 (95% CI: 34.4—35.1) and 51.9 (95% CI: 51.5—52.4) prior to surgery. The median TWL was 32.7% (Q1: 24.5; Q3: 40.7). For the vast majority of patients, the bariatric intervention was either a Roux-en-Y gastric bypass (RYGB, 50%) or sleeve gastrectomy (43%); 5.5% had a gastric band, 0.3% reported gastric balloons and 1.1% reported other procedures.

### AUDs by ICD-10 diagnoses

**Fig. 1:**
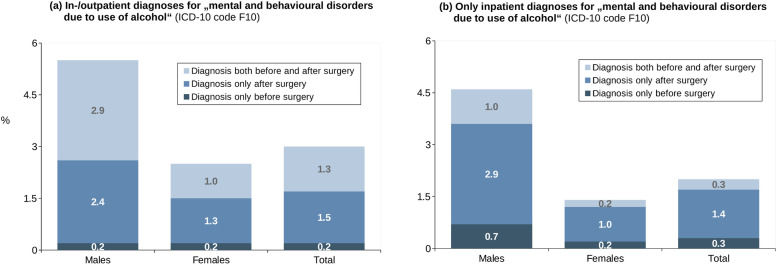
Alcohol use disorders in the patient sample. ICD-10 diagnoses of alcohol use disorders (F10) in the health claims data of the patients, considering patients (**a**) in- and/or outpatient diagnoses or (**b**) at least one inpatient diagnosis (*N* = 2151).

Figure 1 displays the proportion of patients with pre-/post-surgical AUD diagnoses in the health claims data by considering in-/outpatient diagnoses (Fig. 1a) or at least one inpatient diagnosis (Fig. 1b). Overall, 3% of all patients were classified as having an AUD according to claims data (at least one inpatient diagnosis: 2%). AUD diagnoses were 2.2-times more common in men; this difference by sex increased if patients with at least one inpatient diagnosis codes were considered. For both sexes, more than 40% of diagnoses were coded after but not before the surgery. This proportion was 70% if considering only patients with at least one inpatient AUD diagnosis.

### AUDs by AUDIT screening test

For 655 patients, an AUDIT total score could not be computed due to missing of at least one item (predominantly regarding the amount of regular drinking, n = 626). Thus, a total score was available for *n* = 1496 patients. Patients with incomplete AUDITs were older than those with completed AUDITs (55.7 years, 95%-CI: 54.9—56.5 vs. 53.9 years, 95%-CI: 53.4—54.5) and more often male (20.5% vs. 16.8%). When considering the single AUDIT items, non-completers tended to respond more often with lower scores (i.e. “never or less than monthly”, “no”) than completers (see Suppl. Table S2 for more details). In a sensitivity analysis, we additionally compared the AUDIT score distributions after replacing the missing values of the item on the drinking amount by the highest scores (see Supplementary Table S3).

**Fig. 2:**
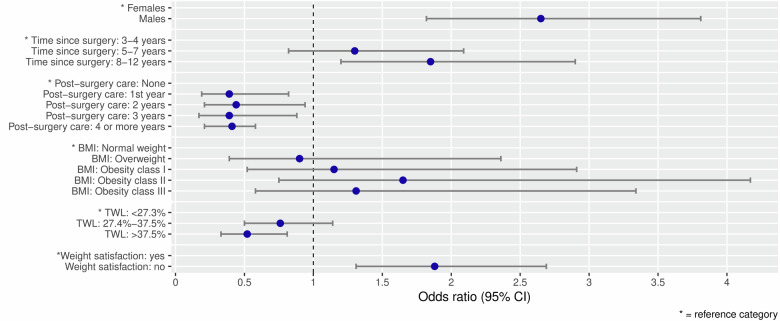
Odds for alcohol use disorders. Associations between patient characteristics and at least a “hazardous consumption of alcohol” (AUDIT score ≥8), estimated by separate logistic regression models and adjusted for sex (*N* = 1496).

The mean AUDIT score was 3.4 (95% CI: 3.1—3.6) with higher scores in men (4.8, 95% CI: 4.2—5.4) than in women (3.4, 95% CI: 2.8—3.2). Regarding type of BS, the mean AUDIT scores were 3.5 (95% CI: 3.2—3.9, “gastric bypass”), 3.2 (95% CI: 2.8—3.5, “gastric sleeve”), 3.0 (95% CI: 3.2—3.0, “gastric band”) and 1.2 (95% CI: 0.2—2.6, “gastric balloon”). Table 1 shows the distribution of the AUDIT categories. Overall, 9.4% of all patients showed at least hazardous/harmful consumption. The proportion was higher in men than women (17.3% vs. 7.3%). In the sensitivity analyses, these proportions increased to up to 18.3% (see Supplementary Table S1), also with higher proportions for men (up to 33.7%) than for women (up to 14.4%). The AUDIT categories did not differ regarding age, time since and type of surgery or BMI (see Table 1). A lower TWL was associated with higher proportions of at least hazardous/harmful consumption. Figure 2 displays the associations between these parameters and an AUDIT score of ≥8, adjusted for sex. A higher likelihood for at least hazardous alcohol consumption was found for males as compared to females, a longer time since surgery and dissatisfaction with post-surgical weight loss. This association still remained stable after additionally adjusting for post-surgical TWL and BMI (OR = 1.74, 95% CI: 1.13–2.68). Post-surgical care of any duration and higher TWLs were associated with a lower likelihood of hazardous drinking.

**Table 1:** Distribution of AUDIT score categories in the patient sample (*N* = 1496).

	*N*	AUDIT score categories (in %)		
		<7	8–14	>15
Total	1496	90.6	5.7	3.7
Male	306	82.7	10.8	6.5
Female	1190	92.7	4.4	2.9
Age groups				
<49 years	500	90.0	6.4	3.6
50–59 years	499	90.8	4.6	4.6
>60 years	497	91.1	6.0	2.8
Time since surgery				
3–4 years	497	93.0	4.2	2.8
5–7 years	484	91.1	5.6	3.3
>8 years	515	88.0	7.2	4.9
Type of bariatric intervention^a^				
Gastric band	71	90.1	7.0	2.8
Gastric bypass	700	90.4	5.9	3.7
Gastric balloon	5	100.0	0.0	0.0
Sleeve	636	91.4	5.5	3.1
Other	15	80.0	13.3	6.7
Body Mass Index^b^				
18.5–24.9 (normal weight)	90	92.2	6.7	1.1
25.0–29.9 (overweight)	334	93.1	4.2	2.7
30.0–34.9 (obesity class I)	410	91.0	5.1	3.9
35.0–39.9 (obesity class II)	318	87.1	7.2	5.7
> 40 (obesity class III)	320	90.0	6.6	3.4
Total weight loss (in %)^c^				
<27.3	484	87.6	7.2	5.2
>27.4–37.5	497	90.5	6.0	3.4
>37.6	483	93.4	3.9	2.7
Satisfaction with weight progression^d^				
Yes	952	92.2	5.3	2.5
No	514	87.4	6.8	5.8

Missing values: ^a^ 96, ^b^ 24, ^c^ 32, ^d^ 30.

**Fig. 3:**
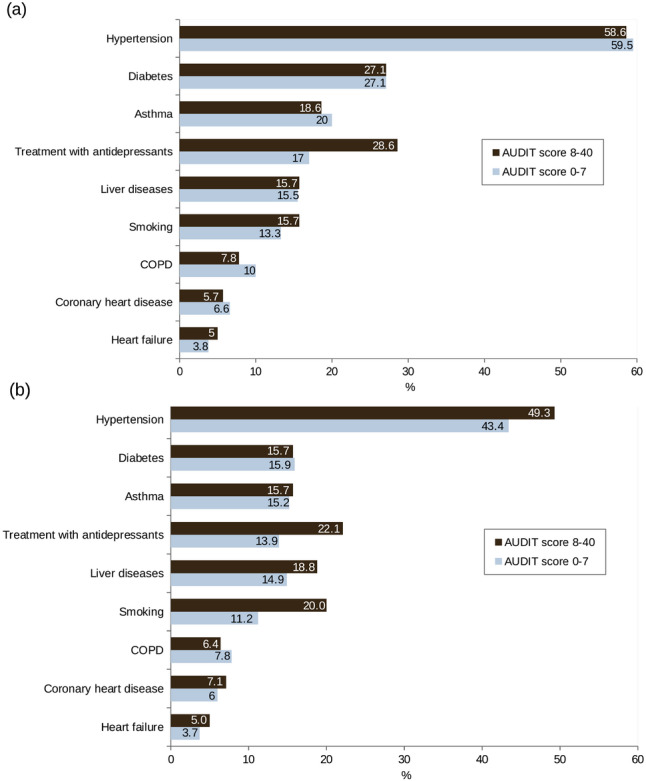
Comorbidities in alcohol use disorders. Frequency of comorbidities as coded at least once during (**a**) baseline and (**b**) follow-up, stratified by AUDIT-Score (0–7: “no alcohol use disorder” vs. ≥8: at least “hazardous consumption”) (*N* = 1496).

For patients with at least hazardous/harmful alcohol consumption according to the AUDIT, also higher numbers of inpatient treatment days and hospitalizations were observed (Table 2) as compared to patients with low-risk consumption. Regarding psychotherapy, the proportion of patients with at least one corresponding reimbursement code were similar across the AUDIT categories. More patients with moderate or severe AUDs reported psychotherapy consultations after surgery as compared to patients with less severe drinking patterns. The proportion of patients with comorbidities during baseline and follow-up is shown in Fig. 3. While overall obesity-related comorbidities (e.g. hypertension, diabetes) decreased post-surgery, there were similar proportions of affected patients among those with higher and those with lower AUDIT-scores, respectively. The only exceptions were antidepressant treatment and smoking, both of which were pre- and post-surgically more common in patients with AUDIT scores >7 than lower scores.

**Table 2:** Health care resources utilization among bariatric patients, stratified by severity of alcohol use disorder according to AUDIT (*N* = 1496).

		AUDIT Score (group)		
		0–7 (*N* = 1 356)	8–14 (*N* = 85)	15–40 (*N* = 55)
**Number of inpatient treatment days**				
During baseline	Median (Q1; Q3)	5 (1; 17)	7 (1; 14)	12 (1.5; 31)
	95% CI	5–6	4–10	2–20
During first year	Median (Q1; Q3)	7 (5; 11)	7 (5; 12)	8 (5; 11)
after surgery	95% CI	7–7	6–9	6–8
During follow-up	Median (Q1; Q3)	13 (6; 35)	19 (9; 48)	17 (8; 42.5)
	95% CI	12–15	12–32	10–25
**Number of hospitalizations**				
During baseline	Median (Q1; Q3)	2 (1; 3)	2 (1; 4)	2 (1; 4)
	95% CI	2–2	2–3	2–3
During first year	Median (Q1; Q3)	1 (0; 2)	1 (0; 2)	1 (0; 1)
after surgery	95% CI	1–1	1–1	0–1
During follow-up	Median (Q1; Q3)	2 (1; 5)	3 (1; 6)	3 (1; 6)
	95% CI	2–2	2–5	2–4
**Number of outpatient visits**				
During baseline	Median (Q1; Q3)	84.5 (58; 121)	78 (61; 125)	85 (51; 111.5)
	95% CI	81–88	73–91	63–96
During first year	Median (Q1; Q3)	30 (19; 43)	25 (17; 44)	28 (18.5; 44)
after surgery	95% CI	28–31	21–33	21–38
During follow-up	Median (Q1; Q3)	89 (38; 177)	123 (43; 222)	117 (58; 185)
	95% CI	80–96	78–157	103–145
**Proportion of patients with psychotherapy health claims, N (%)**				
During baseline		359 (26.5)	25 (29.4)	16 (29.1)
During follow-up		307 (22.6)	23 (27.1)	14 (25.5)
**Proportion of patients reporting psychological counseling, N (%)**				
During follow-up		470 (38.2)	31 (39.7)	32 (60.4)

## Discussion

We investigated the frequency of AUDs in patients after BS based on a large patient sample with a post-bariatric history of up to twelve years by analyzing survey data as well as health claims data from the same patient population. This approach enabled us to at least partially compensate for the inherent disadvantages of one data source (health claims data: no information on lifestyle or subthreshold diseases; survey data: e.g. inherent potential recall bias, inaccuracy of medical information) with the strengths of the other data source (health claims data: e.g. accurate depiction of comorbidities and cross-sectoral health care resource utilization over a long observation period; survey data: e.g. documentation of lifestyle behaviors, trend in post-surgical body weight, evaluation of subthreshold mental disorders). This helped us to get a more complete picture of the research subject.

Based on health claims data, we found a proportion of 2–3% who had been diagnosed with AUDs at least once during the observation period. According to AUDIT, one in ten patients reported risky drinking, with almost six percent meeting the criteria for hazardous alcohol consumption and almost four percent meeting the criteria for alcohol dependence. Almost twice as higher proportions were found when increasing the weighting or the scoring of the AUDIT items that relate to the consumption patterns of alcohol, as well as decreasing the original cut-off scores of the instrument. According to these sensitivity analyses which have been introduced as to our knowledge the AUDIT has not been validated in post-bariatric patients before, one in three male patients and one in seven female patients revealed at least risky consumption patterns.

The performance of our study participants is comparable to previous findings reported from the general population and from patients attending to general practices [25, 26]. Unlike the AUDIT screening, which was applied only once in our study after surgery, the use of health claims data allowed us to count the frequencies of AUDs pre-/post-surgery. Notably, up to 70% of patients with AUDs were diagnosed post-surgery but not before. This is in line with previous studies which reported an increased risk of incident AUDs after BS in patients without an AUD history, based on established psychometric instruments [8, 12, 27, 28]. However, we wish to emphasize that the interpretation of AUDs based on health claims data warrants great caution, since miscodings cannot be fully excluded. While inpatient discharge diagnoses usually can be considered valid, the accuracy of single outpatient diagnoses can be questionable. We attempted to overcome this shortcoming by requiring outpatient diagnoses to be confirmed by at least another outpatient diagnosis (either consecutive or from different physicians). Nonetheless, we attribute a higher diagnostic validity to the group of patients with at least one inpatient diagnosis available in our data than to the group with outpatient diagnoses without the necessity of an inpatient diagnosis. However, it must be clearly stated that our results are of a purely descriptive nature and that a direct, causal relationship between bariatric surgery and the occurrence of AUDs cannot be assessed with our study design. The lack of an otherwise comparable control group but without bariatric intervention is a clear limitation of our study.

In essence, the frequencies we found for severe alcohol disorders are of the same order of magnitude as those reported for non-bariatric populations. In a German nationwide health study, Jacobi et al. [29] found a 12-month prevalence for alcohol dependence of 3%, based on a structured clinical interview (with men affected about four times more often than women). Similarly, in a re-analysis of several European epidemiology studies, Wittchen et al. [30] reported a 12-month prevalence of 3.4% for alcohol dependence. It should be considered, however, that these figures were derived from the general population. In contrast, our data refer to a population for whom AUDs are actually an exclusion criterion for BS and for whom reduced alcohol consumption (if not abstinence) is indicated after surgery [7]. Thus, these figures still give rise to concern. In this context, however, it is remarkable that, although the frequency of alcohol dependence is comparable, the proportion of those without manifest dependence yet with latent alcohol abuse is three times higher than in the general population [29]. That is, although patients in our sample were not more often alcohol dependent than the general population according to the AUDIT, the proportion of patients with “risky drinking” was substantially increased which might indicate that BS puts patients at risk of substance abuse (although, as mentioned before, this cannot be verified with our study design). The incorporation of our study results into the previously reported frequencies of post-bariatric AUDs is complicated due to the methodological heterogeneity of the studies. A recently published review which included 18 articles revealed inconclusive findings with studies reporting both worsening and improvement of drinking behaviors after surgery [31]. Svensson et al. [32] compared the development of AUDs in bariatric patients and in non-bariatric controls with obesity in a prospective cohort study. Almost seven percent of patients after bariatric interventions reported an alcohol consumption pattern beyond low risk, which is slightly lower than our estimates in the main analyses and corresponds to our estimates in the sensitivity analyses, which replaced missing values on the item regarding the quantity of drinks. However, while this work is among the few with a longer follow-up, these results have limited comparability with ours because they are derived exclusively from inpatient data and from patients without a pre-surgical history of AUDs, potentially limiting the generalizability of their findings. Suzuki et al. [33] reported AUDs two times more often (12%) in inpatients after BS, also using the AUDIT instrument for the detection of alcohol disorders, and they found no association between post-surgical weight loss and AUDs. However, patients with a history of AUDs and specific types of surgery were more likely to have post-surgical AUDs. The authors themselves acknowledge that the precision of their study is limited by the small number of patients included (*n* = 51). The validity might have been compromised by a low response rate (11–22%), which potentially introduced bias to their study. According to structured clinical diagnostic interviews of 200 patients up to three years after a RYGB, eight percent developed an AUD and almost half of these patients had no pre-surgical history of AUDs [12]. Similar results were reported for other addictive disorders covered by the interviews. Also, when comparing pre- and post-surgical prevalence rates of high-risk drinking according to the AUDIT-C instrument, Wong et al. [28] reported an almost two times higher proportion of affected patients one year after surgery (23% vs. 13%). Another recently published study investigating AUDs in patients after bariatric surgery by using the AUDIT-C was conducted by White et al. [34]. Based upon a multicenter prospective cohort study on 217 adolescent patients (aged 13—19 years) undergoing BS, the authors concluded that nearly half of all patients screened positive for AUDs during the follow-up of up to eight years. While this study is among the few covering a longer observation period, these findings are difficult to align with ours due to the different age ranges under study. However, especially regarding the age range covered by White and colleagues, which is of high public health relevance, these findings are important and point out to further studies.

We found higher rates of AUDs in patients whose surgery had occurred a longer time ago compared with patients with a shorter post-surgical history. While this finding has to be treated with caution since longer observation periods per se allow a higher cumulative incidence, this result is consistent with several studies suggesting a slow rather than a rapid development of AUDs over several years after surgery, putting emphasis on the need for long-term follow-up and care. For instance, results from a previously published multicenter cohort study covering two thousand patients after BS and a maximum follow-up of seven years [9], prevalence of substance disorders, including AUDs, ranged between 7% at baseline and up to 16% seven years after surgery. Notably, the prevalence as well as the cumulative incidence of AUDs varied by type of bariatric intervention and AUDs were more than twice as high for patients with RYGB as for patients with gastric banding. Again, no data were available for patients with sleeve gastrectomy, as at the time of study conduction this intervention was less common than it is today. As more than 40% of our patients had this type of intervention, our study provides further insights.

However, while the aforementioned studies generally suggest a steady increase in AUDs after surgery, there is also evidence of a more uneven or even reversible course. Wee et al. [35] presented findings from a multicenter study on 375 patients with a post-surgical follow-up of two years. In addition to a proportion of 7% of patients with incident high-risk drinking, more than half of patients with pre-surgical high-risk drinking discontinued this behavior after surgery. Similarly, findings from a re-analysis of ten studies on the risk of post-surgical AUDs demonstrated an increased risk from the third year on but not during the first two years after surgery [13]. However, these findings warrant caution as the majority of the considered studies included comparably fewer participants (eight studies covered 800 patients or fewer), had shorter durations (three years or less in six studies) or offered cross-sectional data only (four studies). The authors emphasize the need for long-term investigations to determine if there is a true increase in AUD prevalence in the context of BS. Similarly, Sen et al. [36] investigated the post-bariatric risk of AUDs as estimated with AUDIT in 183 patients up to 6 years after sleeve gastrectomy and reported a reduction in AUDIT scores in the first 3-year follow-up and an increase in the 4–6-year follow-up. Therefore, regarding the sample size and the long-term follow-up in our study, our data might contribute to these findings. In this regard, it is a limitation that we used the AUDIT screening only once after surgery. Thus, we could not determine a change of drinking patterns with this instrument. However, with all due caution the results from the health claims data discussed earlier might hint at an increasing rather than a declining trend.

We found increased health care resource utilization in patients with risky drinking, even in those patients who showed hazardous consumption but not (yet) alcohol dependency. This held true for the number of hospitalizations as well as for the number of inpatient days. Interestingly, for both measures, these differences only emerged from the second year after surgery, but not during the first year or before. This supports the previously mentioned studies, which suggest a slow rather than an immediate development of AUDs. It is important to note that these differences in our data cannot be explained by higher comorbidities. Although some obesity-related comorbidities (e.g. hypertension, diabetes) were less frequent after surgery than before, the comorbidities between patients with low-risk consumption and hazardous alcohol consumption/AUDs were largely comparable at both points in time. This was true with the exception of smoking and treatment with antidepressants, which can be explained by a higher association of AUDs with depressive disorders and which has been reported in the literature [37]. Against this background, it is noteworthy that according to health claims data, patients with AUDs do not appear to receive or seek psychotherapy more often than patients without. Undertreatment of AUDs, although already a known problem [38], would be of particular concern in this patient population for whom close follow-up care is indicated. However, in the questionnaire, patients with AUDs reported being treated by psychiatrists or psychotherapists more often. This obvious contradiction in our data cannot be satisfactorily resolved. It is not unlikely that laypersons do not know the difference between psychiatrists and psychotherapists. However, since in Germany, the services of both professions can be reimbursed equally by statutory health insurance providers, this unawareness would not explain the difference.

This study was the first to investigate AUDs in patients after BS using survey data and health claims data from the same study population. In addition to the long post-surgical period of up to twelve years, the large sample size also facilitated stratification by sex, enabling us to investigate AUDs in male patients also. This is important, since on the one hand, women with BS surgery clearly outrank men in sample size in most studies, while on the other hand in most populations, alcohol-related disorders are more common in men. Moreover, the use of health claims data—available from each patient irrespective of survey response status—also allowed us to assess whether non-responders and responders differed in terms of sociodemographic and clinical parameters, which was not the case [17]. It can therefore be assumed that the problem of responder bias, which occurs very frequently in field studies, was considerably lower in this study. In addition to the need for caution in the interpretation of our health claims data, as already discussed, a further limitation is that only the F-diagnoses were used. We did not use diagnoses that indicate consequences of AUDs or severe drinking (e.g. fat liver, liver cirrhosis). Furthermore, it can generally be assumed that the extent of alcohol disorders is significantly underestimated in both data sources: only cases of diagnosed AUDs are documented in the health claims data, while clinically significant but not yet full-blown AUDs cannot be identified here. In the survey data, an underestimation can occur due to untrue statements about drinking behavior on the part of the participant and/or a lack of understanding of the “drinking units” as queried in the AUDIT. Against this background, the prevalences we found must be critically scrutinized. This also holds true as the standard cut-off score of the AUDIT might underestimate the true prevalence since it has not been validated for this specific population, which might require lower cut-off recommendations due to altered metabolism of alcohol. Another methodological limitation stems from the gap of at least three years between BS and the survey, as only patients with BS between 2004 and 2018 could be included, but the survey was conducted in 2021. Therefore, it is not possible to use the AUDIT to infer drinking patterns during the first two years after surgery. However, in the context of previous research, we estimate this limitation to be minor because, as discussed earlier, many studies already considered the early years after surgery.

In conclusion, the proportion of patients with BS and with alcohol disorders gives rise to concern as alcohol consumption should be restricted after BS. The results suggest the necessity for close monitoring and post-surgical care.

## Supplementary information

Supplementary Information

Suppl Table S1

Suppl Table S2

Suppl Table S3

Suppl Figure S1

## Data Availability

As we are not the owners of the data we are not legally entitled to grant access to the data of the German Pharmacoepidemiological Research Database. In accordance with German data protection regulations, access to the data is granted only to employees of the Leibniz Institute for Prevention Research and Epidemiology—BIPS on the BIPS premises and in the context of approved research projects. Third parties may only access the data in cooperation with BIPS and after signing an agreement for guest researchers at BIPS.
