# Supplementary material for: Alcohol use disorders after bariatric surgery: a study using linked health claims and survey data
Source: Int J Obes (Lond). 2024 Sep 6;48(11):1656–63. doi: 10.1038/s41366-024-01606-3 (PMC11502494; doi:10.1038/s41366-024-01606-3)
Supplement: Supplementary file 1 — Supplementary Information [file 41366_2024_1606_MOESM1_ESM.docx]

**Supplementary Information**

**Suppl Table S1:** Sensitivity analyses for the proportion of patients with low-risk/hazardous alcohol consumption or moderate to severe alcohol use disorders.

**Suppl. Table S2:** Comparison of the responses to the AUDIT-items (except frequency and amount of drinking) between completers (N=1 496) and non-completers (N=655) of the AUDIT.

**Suppl. Table S3:** AUDIT total score distributions when replacing the missing values (n=626) of the item *“How many standard drinks containing alcohol do you have on a typical day when drinking?”*

**Suppl. Figure S1:** Patient ascertainment.
