# Supplementary material for: Alcohol use disorders after bariatric surgery: a study using linked health claims and survey data
Source: Int J Obes (Lond). 2024 Sep 6;48(11):1656–63. doi: 10.1038/s41366-024-01606-3 (PMC11502494; doi:10.1038/s41366-024-01606-3)
Supplement: Supplementary file 2 — Suppl Table S1 [file 41366_2024_1606_MOESM2_ESM.docx]

| **Suppl. Table S1:** | Sensitivity analyses for the proportion of patients with low-risk/hazardous alcohol consumption or moderate to severe alcohol use disorders. |
| --- | --- |

|  | **Sensitivity analysis** | | |
| --- | --- | --- | --- |
|  | **(A)**^1^ | **(B)**^2^ | **(C)**^3^ |
| **Total, N (%)** |  |  |  |
| AUDIT category: „low-risk consumption“ | 1 247 (83.4) | 1250 (83.5) | 1 222 (81.7) |
| AUDIT category: „hazardous/harmful consumption“ | 174 (11.6) | 176 (11.8) | 196 (13.1) |
| AUDIT category: „moderate to severe AUD“ | 75 (5.0) | 70 (4.7) | 78 (5.2) |
|  |  |  |  |
| **Males, N (%)** |  |  |  |
| AUDIT category: „low-risk consumption“ | 213 (69.6) | 214 (69.9) | 203 (66.3) |
| AUDIT category: „hazardous/harmful consumption“ | 65 (21.2) | 66 (21.6) | 75 (24.5) |
| AUDIT category: „moderate to severe AUD“ | 28 (9.2) | 26 (8.5) | 28 (9.2) |
|  |  |  |  |
| **Females, N (%)** |  |  |  |
| AUDIT category: „low-risk consumption“ | 1 034 (86.9) | 1 036 (87.1) | 1 019 (85.6) |
| AUDIT category: „hazardous/harmful consumption“ | 109 (9.2) | 110 (9.2) | 121 (10.2) |
| AUDIT category: „moderate to severe AUD“ | 47 (3.9) | 44 (3.7) | 50 (4.2) |

AUD = alcohol use disorder

^1^ Double weight for the AUDIT consumption items (questions 1-3);

^2^ Adding +1 to each score of the AUDIT consumption items (questions 1-3)

^3^ Reducing the cut-off scores to 0-4 („low-risk consumption“), 5-11 („hazardous/harmful consumption“) and 12-40 („moderate to severe AUD“)
