# Supplementary material for: Alcohol use disorders after bariatric surgery: a study using linked health claims and survey data
Source: Int J Obes (Lond). 2024 Sep 6;48(11):1656–63. doi: 10.1038/s41366-024-01606-3 (PMC11502494; doi:10.1038/s41366-024-01606-3)
Supplement: Supplementary file 3 — Suppl Table S2 [file 41366_2024_1606_MOESM3_ESM.docx]

**Suppl. Table S2:** Comparison of the responses to the AUDIT-items (except frequency and amount
 of drinking) between completers and non-completers of the AUDIT

|  |  | **Completer**  **(N = 1,496)** | | **Non-completer**  **(N = 655)** | |  |
| --- | --- | --- | --- | --- | --- | --- |
|  |  | **Count** | **%** | **Count** | **%** |  |
|  |  |  |  |  |  |  |
| **“How often do you have a drink containing alcohol?”** | | | | | | |
|  | Never | 81 | 5.4 | 586 | 89.5 |  |
|  | ≤ 1x per month | 724 | 48.4 | 29 | 4.4 |  |
|  | 2–4x per month | 445 | 29.7 | 15 | 2.3 |  |
|  | 2–4x per week | 165 | 11.0 | 9 | 1.4 |  |
|  | ≥ 4x per week | 81 | 5.4 | 3 | 0.5 |  |
|  | Missing value |  |  | 13 | 2.0 |  |
|  |  |  |  |  |  |  |
| **“How often do you have six or more drinks on one occasion?”** | | | | | | |
|  | 1 to 2 | 982 | 65.6 | 589 | 89.9 |  |
|  | 3 to 4 | 328 | 21.9 | 15 | 2.3 |  |
|  | 5 to 6 | 111 | 7.4 | 4 | 0.6 |  |
|  | 7 to 9 | 52 | 3.5 | 7 | 1.1 |  |
|  | 10 or more | 23 | 1.5 | 0 | 0.0 |  |
|  | Missing value |  |  | 40 | 6.1 |  |
|  |  |  |  |  |  |  |
| **“During the past year, how often have you found that you were not able to stop drinking once you had started?”** | | | | | | |
|  | Never | 1,377 | 92.0 | 562 | 85.8 |  |
|  | < 1x per month | 54 | 3.6 | 2 | 0.3 |  |
|  | 1x per month | 24 | 1.6 | 1 | 0.2 |  |
|  | 1x per week | 21 | 1.4 | 1 | 0.2 |  |
|  | Daily or almost daily | 20 | 1.3 | 3 | 0.5 |  |
|  | Missing value |  |  | 86 | 13.1 |  |
|  |  |  |  |  |  |  |
| **“During the past year, how often have you failed to do what was normally expected of you because of drinking?“** | | | | | | |
|  | Never | 1,441 | 96.3 | 568 | 86.7 |  |
|  | < 1x per month | 21 | 1.4 | 4 | 0.6 |  |
|  | 1x per month | 17 | 1.1 | 1 | 0.2 |  |
|  | 1x per week | 11 | 0.7 | 0 | 0.0 |  |
|  | Daily or almost daily | 6 | 0.4 | 3 | 0.5 |  |
|  | Missing value |  |  | 79 | 12.1 |  |
|  |  |  |  |  |  |  |
| **“During the past year, how often have you needed a drink in the morning to get yourself going after a heavy drinking session?”** | | | | | | |
|  | Never | 1,471 | 98.3 | 577 | 88.1 |  |
|  | < 1x per month | 14 | 0.9 | 1 | 0.2 |  |
|  | 1x per month | 4 | 0.3 | 0 | 0.0 |  |
|  | 1x per week | 4 | 0.3 | 0 | 0.0 |  |
|  | Daily or almost daily | 3 | 0.2 | 0 | 0.0 |  |
|  | Missing value |  |  | 77 | 11.8 |  |
|  |  |  |  |  |  |  |
| **“During the past year, how often have you had a feeling of guilt or remorse after drinking?”** | | | | | | |
|  | Never | 1,361 | 91.0 | 567 | 86.6 |  |
|  | < 1x per month | 57 | 3.8 | 4 | 0.6 |  |
|  | 1x per month | 30 | 2.0 | 0 | 0.0 |  |
|  | 1x per week | 18 | 1.2 | 0 | 0.0 |  |
|  | Daily or almost daily | 30 | 2.0 | 5 | 0.8 |  |
|  | Missing value |  |  | 79 | 12.1 |  |
|  |  |  |  |  |  |  |
| **“During the past year, how often have you been unable to remember what happened the night before because you had been drinking?”** | | | | | | |
|  | Never | 1,404 | 93.9 | 568 | 86.7 |  |
|  | < 1x per month | 58 | 3.9 | 2 | 0.3 |  |
|  | 1x per month | 16 | 1.1 | 0 | 0.0 |  |
|  | 1x per week | 11 | 0.7 | 0 | 0.0 |  |
|  | Daily or almost daily | 7 | 0.5 | 3 | 0.5 |  |
|  | Missing value |  |  | 82 | 12.5 |  |
|  |  |  |  |  |  |  |
| **“Have you or someone else been injured as a result of your drinking?”** | | | | | | |
|  | No | 1,460 | 97.6 | 597 | 91.1 |  |
|  | Yes, but not in the last year | 24 | 1.6 | 11 | 1.7 |  |
|  | Yes, during the last year | 12 | 0.8 | 0 | 0.0 |  |
|  | Missing value |  |  | 47 | 7.2 |  |
|  |  |  |  |  |  |  |
| **“Has a relative or friend, doctor or other health worker been concerned about your drinking or suggested you cut down?”** | | | | | | |
|  | No | 1,403 | 93.8 | 576 | 87.9 |  |
|  | Yes, but not in the last year | 29 | 1.9 | 11 | 1.7 |  |
|  | Yes, during the last year | 64 | 4.3 | 4 | 0.6 |  |
|  | Missing value |  |  | 64 | 9.8 |  |
