# Supplementary material for: Alcohol use disorders after bariatric surgery: a study using linked health claims and survey data
Source: Int J Obes (Lond). 2024 Sep 6;48(11):1656–63. doi: 10.1038/s41366-024-01606-3 (PMC11502494; doi:10.1038/s41366-024-01606-3)
Supplement: Supplementary file 4 — Suppl Table S3 [file 41366_2024_1606_MOESM4_ESM.docx]

| **Suppl. Table S3.** | AUDIT total score distributions when replacing the missing values (n=626) of the item *“How many standard drinks containing alcohol do you have on a typical day when drinking?”* |
| --- | --- |

|  |  |  |  |  |  |
| --- | --- | --- | --- | --- | --- |
|  |  |  | **AUDIT Score (in %)** | | |
| **Replacement of missing values** | **Median (Q1; Q3)** |  | **<7** | **8–14** | **>15** |
|  |  |  |  |  |  |
| No replacement of missing values | 2 (1; 4) |  | 90.6 | 5.7 | 3.7 |
| Replacement of missing values with “0” | 1 (0; 3) |  | 93.0 | 4.2 | 2.8 |
| Replacement of missing values with “1” | 1 (1; 3) |  | 93.0 | 4.2 | 2.8 |
| Replacement of missing values with “2” | 2 (1;3) |  | 93.0 | 4.2 | 2.8 |
| Replacement of missing values with “3” | 3 (1; 3) |  | 92.9 | 4.3 | 2.8 |
| Replacement of missing values with “4” | 3 (1; 4) |  | 92.7 | 4.5 | 2.8 |
|  |  |  |  |  |  |
