# Supplementary figures and images for: Alcohol use disorders after bariatric surgery: a study using linked health claims and survey data
Source: Int J Obes (Lond). 2024 Sep 6;48(11):1656–63. doi: 10.1038/s41366-024-01606-3 (PMC11502494; doi:10.1038/s41366-024-01606-3)

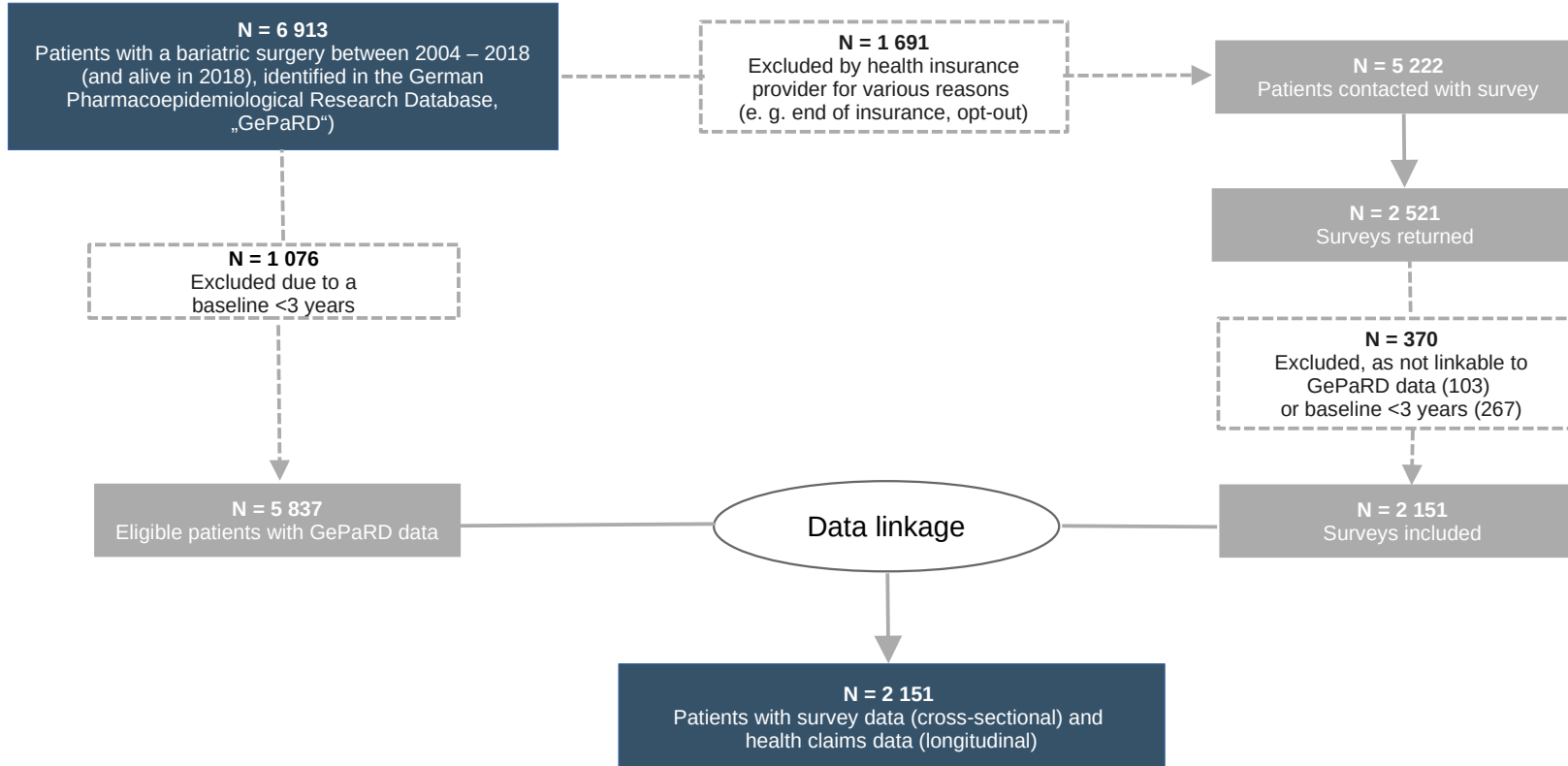

Supplement: Supplementary file 5 — Suppl Figure S1 [file 41366_2024_1606_MOESM5_ESM.pdf]
